# BARIATRIC SURGERY IN BRAZILIAN PUBLIC HEALTH SYSTEM: THE GOOD, THE BAD AND THE UGLY, OR A LONG WAY TO GO. YELLOW SIGN!

**DOI:** 10.1590/0102-672020190001e1470

**Published:** 2019-12-20

**Authors:** Antoninho José TONATTO-FILHO, Felipe Melloto GALLOTTI, Marcio Fernandes CHEDID, Tomaz de Jesus Maria GREZZANA-FILHO, Ana Maria Stapasolla Vargas GARCIA

**Affiliations:** 1 General Surgery, Nossa Senhora da Conceição Hospital, Porto Alegre, RS, Brazil;; 2 Digestive Surgery, Porto Alegre Hospital de Clínicas, Porto Alegre, RS, Brazil.

**Keywords:** Obesity, Bariatric surgery, Gastric bypass, Obesidade, Cirurgia bariátrica, Derivação gástrica

## Abstract

**Background:**

In Brazil, there has been a significant increase in obesity rates in all age groups. Data from 2017 show that obesity affects 19% of the population. Due to the magnitude of the problem, public health policies have aimed to prevent complications related to obesity by increasing the offerfor bariatric surgeries.

**Aim:**

To analyze the current status of bariatric surgery performed in the Brazilian public health system, including data from macroregions and also the effect of digestive surgery training on the number of procedures.

**Methods:**

The database of the public health registry (DATASUS) was assessedbetween 2008 and 2018 for descriptive analysis of data and evaluation of the selected parameters. The main surgical techniques, comorbidities, mortality and the costs profile of the system were evaluated.

**Results:**

There was a 339% increase in the number of bariatric surgeries in the period evaluated. Gastric bypass was performed in 94% of cases whereas sleeve in 2.4%. Other techniques were used in 3.6%. There were discrepancies in the number of surgeries performed in different regions of the country*.*

**Conclusion:**

There was a considerable advance in the number of bariatric surgeries performed by the public health system between 2008 and 2018. However, there is a need to increase the offer of this service and alsospecialized training, as well as a correction in the distribution of these procedures in the national territory to achieve integrality among its users.

## RESUMO

**Racional:**

No Brasil, tem-se observado aumento expressivo nas taxas de obesidade em todas as faixas etárias. Dados de 2017 demonstram que a obesidade atinge 19% da população. Em virtude da magnitude do problema, as políticas de saúde pública têm focado suas atenções na prevenção das complicações relacionadas à obesidade, através de um incremento na oferta de operações bariátricas.

**Objetivo:**

Analisar o atual estado da cirurgia bariátrica realizada pelo sistemapúblico de saúde do Brasil, incluindo dados das macrorregiões e o efeito do treinamento em cirurgia digestiva no número de procedimentos.

**Métodos:**

Foi utilizado o banco de dados do registro de saúde pública (DATASUS) entre 2008 e 2018 para análise descritiva dos dados e avaliação dos parâmetros selecionados. Foram avaliadas as principais técnicas cirúrgicas, as comorbidades, a mortalidade e o perfil de custos ao sistema.

**Resultados:**

Demonstrou-se aumento de 339% no número de operações bariátricas no período avaliado. O bypass gástrico foi utilizado em 94%, osleeve em 2,4% eoutras técnicas em 3,6% dos casos. A técnica videolaparoscópica foi usada em 4,7% do total entre 2017 e 2018. Observaram-se discrepâncias no número de procedimentos realizados nas diferentes regiões do país.

**Conclusão:**

Houve avanço considerável no número de operações bariátricas realizadas pelo sistema público de saúde entre 2008 e 2018. Há, contudo, necessidade de aumento na oferta deste serviço e de treinamento especializado, bem como correção na distribuição dos procedimentos no território nacional para que se atinja integralidade entre seus usuários.

## INTRODUCTION

Obesity is defined by WHO as the abnormal or excessive accumulation of body fat in the form of adipose tissue. It is considered a multifactorial disease, encompassing genetic, behavioral, metabolic and environmental factors^24^. It is directly associated with the risk of developing chronic diseases such as type II diabetes mellitus, cardiovascular disease, dyslipidemia, sleep apnea, osteoarthritis, dental changes and various types of neoplasms^18^ ^,^ ^24^. Initially, nutritional monitoring, physical activity and medication use are recommended. However, when obesity reaches grade III (BMI >40), clinical treatment results are unsatisfactory in 95% of patients, who regain initial weight within two years^21^. Thus, bariatric surgery is the most effective alternative for the treatment of morbid obesity and its complications^13^ ^,^ ^16^ ^,^ ^26^. The main technique currently used is gastric bypass, which reduces the gastric cavity and the amount of food ingested (restrictive), while at the same time decreasing the intestinal absorption surface (disabsorptive). Its advantages are good metabolic outcome, weight loss and increased satiety, the latter due to the effect on ghrelin secretion, a hormone linked to satiety^5^ ^,^ ^25^. It is the operation of choice for diabetics as it increases the secretion of GLP-1 (glucagon-like peptide-1). Among its disadvantages are higher morbidity and mortality, anemia, vitamin deficiency, hypoproteinemia, and anatomical changes that put more difficulties but do not hinder endoscopic bile duct procedures^10^.

More recently, sleeve gastrectomy has gained popularity because it has a favorable metabolic outcome, adequate weight loss and lower nutritional disorders compared to gastric bypass. Its main disadvantage is the increased incidence of gastroesophageal reflux. It also decreases ghrelin and increases GLP-1 secretion. Other techniques are being indicated less frequently or progressively replaced. Depending on the technique used, this decrease may be attributed to a lower antidiabetic effect, less maintenance of lost weight or greater deleterious metabolic effects. The indications, according to the resolution of the Brazilian Federal Council of Medicine^3^ ^,^ ^4^ are for adult patients with resistance to clinical treatment for at least two years and with BMI >40 or BMI >35 associated with life-threatening comorbidities. Adolescents aged 16 to 18 years can be operated provided that there is pediatric follow-up in the multidisciplinary team and respected the consolidation of wrist cartilage epiphyses. The latest Council Resolution (2,172/2017) expanded the indication for bariatric surgery for patients with type II diabetes mellitus ranging from 30-70 years and BMI from 30-34.9, provided that the disease was not controlled with clinical treatment and diagnosis have been defined for at least 10 years. The contraindications for bariatric surgery are alcohol or illicit drug dependence, uncontrolled severe psychiatric disease, moderate to severe dementia, unstable coronary artery disease, and severe coagulopathy and/or heart disease^4^ ^,^ ^5^.

The aim of this study was to analyze the current state of bariatric surgery performed by the Brazilian public health system, including macroregion data and the effect of digestive surgery training on the number of procedures.

## METHODS

This study was approved by the Institutional Research Ethics Committee under number 5133571821. It is a cross-sectional evaluation with prevalence analysis conducted between January 2008 and December 2018. Data were extracted from DATASUS (Brazilian Public Health Registry Database)^14^. The affiliated hospitals that are part of the Unified Health System (SUS) use a Hospitalization Authorization, which identifies the procedure to be performed, the proposed treatment and the payment that will be made later by the government. Data for gastric sleeve procedures were included only from 2013 and laparoscopic ones from 2017, when these procedures were first computed in the system.

## RESULTS

**FIGURE 1:**
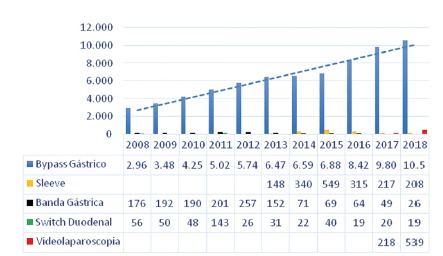
Brazilian SUS bariatric operations from 2008 to 2018

**FIGURE 2:**
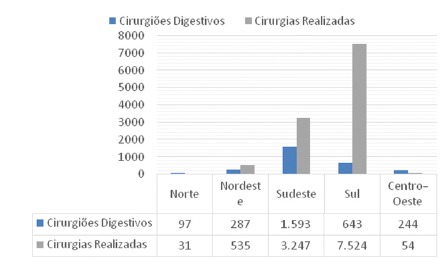
Number of digestive surgeons and bariatric operations in 2018

**FIGURE 3:**
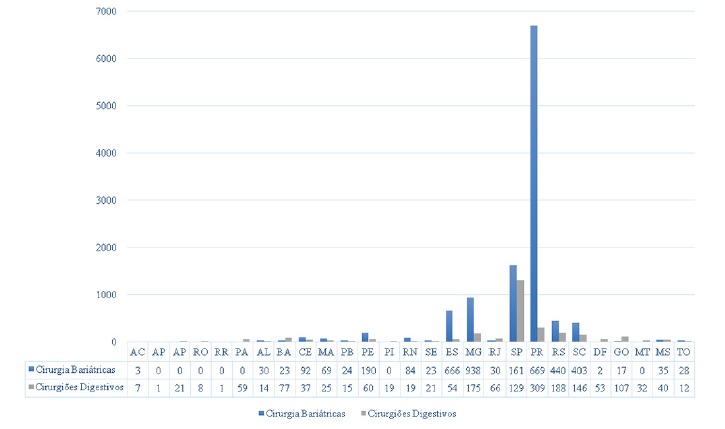
Distribution of the number of bariatric operations and digestive surgeons by state

**FIGURE 4:**
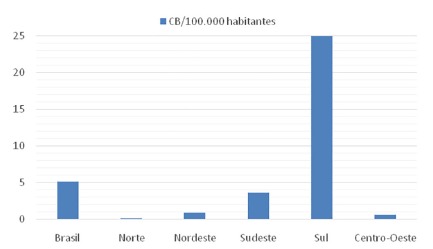
Variation in bariatric operations/inhabitants by region

The four main bariatric techniques and their respective frequencies are shown in Table 1. There was a predominance of gastric bypass in the years evaluated, accounting for 94.9% of the procedures, while the other techniques were performed less frequently: vertical gastrectomy (2.4%), vertical banded gastroplasty (1.9%) and duodenal switch (0.6%). The graphical representation of the increase in operations in the evaluated period, year by year, is shown in Figure 1. There was a progressive increase in the number of procedures performed between 2008-2018, with 3,195 bariatric operations performed in 2008, while in 2018 were 10,852, with 339% growth. Gastric bypass was the most commonly performed procedure in all regions of the country during this period, representing 92% of total operations in 2008 and 97% in 2018. Regionally, the Southern region had the highest absolute number during the period evaluated with 41,764 (56%) operations, followed by the Southeast region, with 25,147 (34%). The Northeast, Midwest and North regions performed, respectively, 5,231 (7%), 1,097 (1.4%) and 707 (0.9%) procedures. The region with the largest increase in the period was the Southern region, with 505%, followed by the Southeast, with 246%. The Northeast, North and Midwest regions showed a decrease in the number of operations. When analyzing the density of bariatric surgery performed throughout the territory of Brazil by the public health system based on the population of 2018^9^, the average of procedures was 5.2 bariatric operations/100,000 inhabitants, as shown in Figure 4. The South region showed the best performance with 25 bariatric operations/100,000 inhabitants, followed by the Southeast regions with 3.7; Northeast with 0.9; Midwest with 0.68 and North with 0.17. Data on the number of laparoscopic procedures distributed by region are shown in Table 2. It was observed that in the short observation period (2017-2018), there was an increase in this technique, and the Northeast region presented the largest percentage increase of operations in the period (246%). However, there was also an increase in all regions. The number of bariatric surgeries according to the number of digestive surgeons is shown in Figure 2. The distribution according to the federative entities is shown in Figure 3. When analyzing the distribution of the number of digestive surgeons among the five national regions, between 2011^19^ and 2018^20^ the Southeast region remained with the highest absolute numbers, with 726 in 2011 and 1,593 in 2018 (representing 55.6% of the total digestive surgeons last year), followed in 2018 by the regions South with 643 (22.4%), Northeast 287 (10%), Midwest 244 (8.5%) and North with 87 (3%). Regarding the percentage increase of digestive surgeons by region, the South region presented the most significant increase, with 309% (157 in 2011 to 643 in 2018), followed by the Northeast 282% (75 to 287), Midwest 269% (66 to 244), North 203% (33 to 97) and Southeast 119% (726 to 1,593).

**TABLE 1:** More frequent bariatric procedures in Unified Brazilian Health System from 2008 to 2018^6^

Gastrointestinal gastroplasty (Gastric bypass)	70.248 ( 94.9%)
Sleeve gastrectomy	1.777 (2.4%)
Gastric banding vertical gastroplasty	1.477 (1.9%)
Duodenal bypass vertical gastrectomy (duodenal switch)	474 (0.6%)

**TABLE 2:** Bariatric procedures by Brazilian geographic regions^6^

	Gastric bypass			Sleeve gastrectomy		Gastric banding vertical gastroplasty			Duodenal bypass vertical gastrectomy (duodenal switch)			Video-laparoscopic bariatric operations	
	2008	2013	2018	2013	2018	2008	2013	2018	2008	2013	2018	2017	2018
North	40	94	31	-	-	10	3	-	-	1	-	-	-
Northeast	362	476	251	4	64	13	54	15	-	8	4	58	201
Southeast	1.156	2.331	2.991	48	60	81	26	9	8	8	9	98	178
South	1.295	3.525	7.307	72	84	58	39	2	40	13	6	61	125
Midwest	110	45	19	24	-	14	30	-	8	1	-	1	35
Total	2.963	6.471	10.599	148	208	176	152	26	56	31	19	218	539

## DISCUSSION

Since the 1970s, changes in eating habits, increased physical inactivity, the presence of genetic factors, increased income in classes C and D and consumption of unhealthy products have contributed, among others, to the increase in overweight and obesity in Brazil. A survey conducted in 2017 by the Ministry of Health’s Risk Factors and Chronic Disease Surveillance Sector showed that 19% are obese and that more than half of this population (54%) are overweight^2^. Another alarming fact is the progression of obesity in the young population, and in ten years (2007-2017) there has been an increase in obesity rates of 110% in the number of people between 18 and 24 years old, almost double the increase in all ranges age groups (60%). In the range of 25 to 34 years there was an elevation of 69%; from 35 to 44 years 23%; from 45 to 54 years 14%; from 55 to 64 years16%; and in the elderly over 65 years there was a 2% growth^1^. Taking into account the entire population, the percentage of severe grade III obese individuals with BMI between 40 and 50 kg/m^2^ reached 0.7% and of superobese with BMI=50 kg/m^2^ was 0.04%. In absolute numbers there are over one million people with severe obesity in the country, affecting mainly women^15^.

Worldwide evidence shows that in 2015 obesity contributed to four million deaths, representing 7.1% of all deaths from any cause^7^. On the other hand, recent data have shown that patients undergoing bariatric surgery have a 33% lower risk of developing any cancer than those with severe obesity who have not undergone surgery. Comparing the results of bariatric surgery with clinical treatment, surgical treatment produces a greater reduction in body weight, a drop in serum glycated hemoglobin, triglyceride and cholesterol levels, a reduction in the need for insulin and cardiovascular medications, and an increase in quality of life^17^. In addition, patients undergoing bariatric surgery had control of hypertension in 51% of cases^8^. Systematic review that evaluated the profile of patients undergoing bariatric surgery assisted by SUS showed that, on average, they are 41.4 years old and have a BMI of 48.6. Of the total, 79% are women, 61% hypertensive, 22% diabetic and 31% have sleep apnea^11^. Compared with patients included in international studies, Brazilians have similar anthropometric and comorbidities, except for the higher prevalence of hypertension. Our data show that the number of surgical procedures performed by SUS in the evaluated period has advanced in all regions. However, the number of procedures remains far below the need determined by the installed epidemiological picture.

Given that there are over one million severely obese people and that number is likely to increase; large investments in the public sector will be needed to mitigate morbid obesity-related complications in the underprivileged population. It is also observed that there is great disparity between the evaluated regions. These data reveal that the most developed regions of the country concentrate the public offer of this surgical procedure. The data reveal that the South and Southeast regions concentrate 94.5% of the total amount of public system bariatric procedures in Brazil. Despite the significant increase in the number of operations, the Brazilian public health system is far behind private in the surgical treatment of morbid obesity. According to data from the Brazilian Society of Bariatric and Metabolic Surgery^22^, in 2017, 105,642 bariatric operations were performed in the private sector. Already in the public system were 10,089, which represent only 9.5% of the total. This number is more relevant when one has in mind that the absolute majority of the population has only treatment conditions in the public service. A relevant point was the beginning of the availability of data on the use of videolaparoscopy for bariatric surgery by the public system in 2017^23^. In the brief period observed, there was a significant increase in the application of this technique, which has significant postoperative advantages and return to daily activities. Today, videolaparoscopy represents 4.7% of the total procedures performed at SUS. In contrast, other techniques such as banded gastroplasty and the duodenal switch have been gradually reduced or disused. It was also observed that training in bariatric surgery and videolaparoscopic surgery plays a fundamental role in increasing the number of procedures. Thus, the data show that during the period evaluated, the growth of bariatric surgery is directly related to the increase in the number of surgeons who specialize in digestive surgery, who are qualified to perform all available bariatric techniques.

The unequal distribution of these professionals across the country also showed a correlation between specialized training and the number of procedures per region, as observed in Figure 2. The number of perioperative deaths during the study period was 16113, representing 0.22% of the total. The highest rate was observed in the Midwest (0.79%) and the lowest in the Northeast, with (0.11%). We do not have data showing the percentage of deaths awaiting bariatric surgery in Brazil, but the mean waiting time, according to some authors, exceeds four years^14^. Thus, the health risks and costs of delaying definitive treatment, while not measurable by the available data, certainly have a negative impact on this population and the entire system. Investment in this area in the last decade was R$ 451,033,063.85, with a progressive increase over the years. As an example, in 2018, R$ 72,965,381.65 were employed. The average hospitalization value was R$ 6,399.35 per patient when assessed throughout the period^14^.

Obviously bariatric surgery is not the solution to contain the evolution of severe obesity in the country, but an important tool to treat related complications once the advanced picture of this disease is installed. This study allows the guidance of managers and professionals involved in the fight against obesity for the formulation of public policies and the establishment of strategies to increase population coverage. The orientation of public policies to contain the progress of the epidemic obesity must be fundamentally based on the prevention of obesity. Several points concerning the universality of the system, equity and justice need to be reevaluated, as well as the obesity prevention policy since childhood and adolescence.

## CONCLUSION

There was an improvement in the number of bariatric procedures performed in the country in the 2008-2018 decade. However, there is a need for a significant increase in surgeon training and the number of bariatric operations in the coming years. Finally, a policy is needed to allow a better distribution of these procedures in the Brazilian regions.
